# RBioplot: an easy-to-use R pipeline for automated statistical analysis and data visualization in molecular biology and biochemistry

**DOI:** 10.7717/peerj.2436

**Published:** 2016-09-28

**Authors:** Jing Zhang, Kenneth B. Storey

**Affiliations:** Institute of Biochemistry, Departments of Biology and Chemistry, Carleton University, Ottawa, ON, Canada

**Keywords:** R package, Bioinformatics, Biostatistics, Histogram, Joint-point curve

## Abstract

**Background:**

Statistical analysis and data visualization are two crucial aspects in molecular biology and biology. For analyses that compare one dependent variable between standard (e.g., control) and one or multiple independent variables, a comprehensive yet highly streamlined solution is valuable. The computer programming language R is a popular platform for researchers to develop tools that are tailored specifically for their research needs. Here we present an R package RBioplot that takes raw input data for automated statistical analysis and plotting, highly compatible with various molecular biology and biochemistry lab techniques, such as, but not limited to, western blotting, PCR, and enzyme activity assays.

**Method:**

The package is built based on workflows operating on a simple raw data layout, with minimum user input or data manipulation required. The package is distributed through GitHub, which can be easily installed through one single-line R command. A detailed installation guide is available at http://kenstoreylab.com/?page_id=2448. Users can also download demo datasets from the same website.

**Results and Discussion:**

By integrating selected functions from existing statistical and data visualization packages with extensive customization, RBioplot features both statistical analysis and data visualization functionalities. Key properties of RBioplot include:

- Fully automated and comprehensive statistical analysis, including normality test, equal variance test, Student’s t-test and ANOVA (with post-hoc tests);
- Fully automated histogram, heatmap and joint-point curve plotting modules;
- Detailed output files for statistical analysis, data manipulation and high quality graphs;
- Axis range finding and user customizable tick settings;
- High user-customizability.

## Introduction

Molecular biology and biochemistry research heavily depends on the interpretation of the data obtained from various lab techniques, such as western blotting, PCR and a wide range of enzyme activity assays. A fully automated and comprehensive statistical analysis and data visualization package is highly desirable for enhancing data processing efficiency as well as reducing erroneous actions. Due to its relatively smooth learning curve, rich resources and strong community support, the computer programming language R has recently become a popular platform for data science in biological research. With both strong built-in statistical functionality and third-party developed packages such as the data visualization package ggplot2 (Wickham, 2009), as well as the stats package multcomp (Hothorn, Bretz & Westfall, 2008), R has enabled the possibility of developing data analysis packages specifically tailored for molecular biology and biochemistry.

The principal goal of the present R package RBioplot resides in the concept of minimizing user input while presenting a comprehensive statistical and data visualization solution, also with the option of high user-customizability. Therefore, automatic data evaluation and processing is crucial. The package contains a statistical analysis module (core function: rbiostats), a histogram plotting module (core function: rbioplot), a heatmap module (core function: rbioplot_heatmap) a joint-point curve plotting module (core functions: rbioplot_curve), and an axis range finding module (core functions: autorange_bar_y and autorange_curve). For both statistical analysis and data visualization modules, we have developed comprehensive workflows that dynamically combine selected functions from the existing packages with new functionalities specifically designed for the datasets obtained from the most popular lab techniques in molecular biology and biochemistry. As a result, RBioplot conducts statistical analysis and data visualization in a fully automated fashion with detailed reports for statistical analysis and high quality image files as output files.

To minimize user intervention of the raw data and maintain consistency among the modules, all the functions operate on an universal input data format (i.e., file type and data points arrangement). Specifically, the input file is a data matrix (.csv) with rows for the standard and independent variables (raw data, with biological replicates) and columns for the dependent variable. All functions are also capable of properly processing datasets with missing data points with no special action required from the user. Accordingly, RBioplot is applicable to datasets obtained from a wide variety of molecular biology and biochemistry lab techniques, including, but not limited to, western blotting, PCR (both endpoint and quantitative PCRs), ELISA, as well as enzyme activity assays.

## Methods and Models

### Statistical analysis module

The core function for the stats module is rbiostats. The function utilizes Student’s t-test and analysis of variance (ANOVA) with post-hoc tests (e.g., Tukey and Dunnett’s tests) to test significance of changes for two- and multi- group comparisons (Tukey, 1949; Shapiro & Wilk, 1965), respectively. Since both tests are based on normal distribution, Shapiro-Wilk test is used to test data normality for each data group (Dunnett, 1955). Moreover, due to the fact that both Student’s t-test and the conventional ANOVA assume equal variance between replicates among standard and independent variables, a variance homogeneity test is needed. With the Shapiro-Wilk normality test in place, we use Bartlett’s equal variance test for testing variance homogeneity, for which a normal distribution is required (Bartlett, 1937). As such, for each dataset, the stats module tests data normality and homogeneity prior to the significance tests by default.

**Figure 1:**
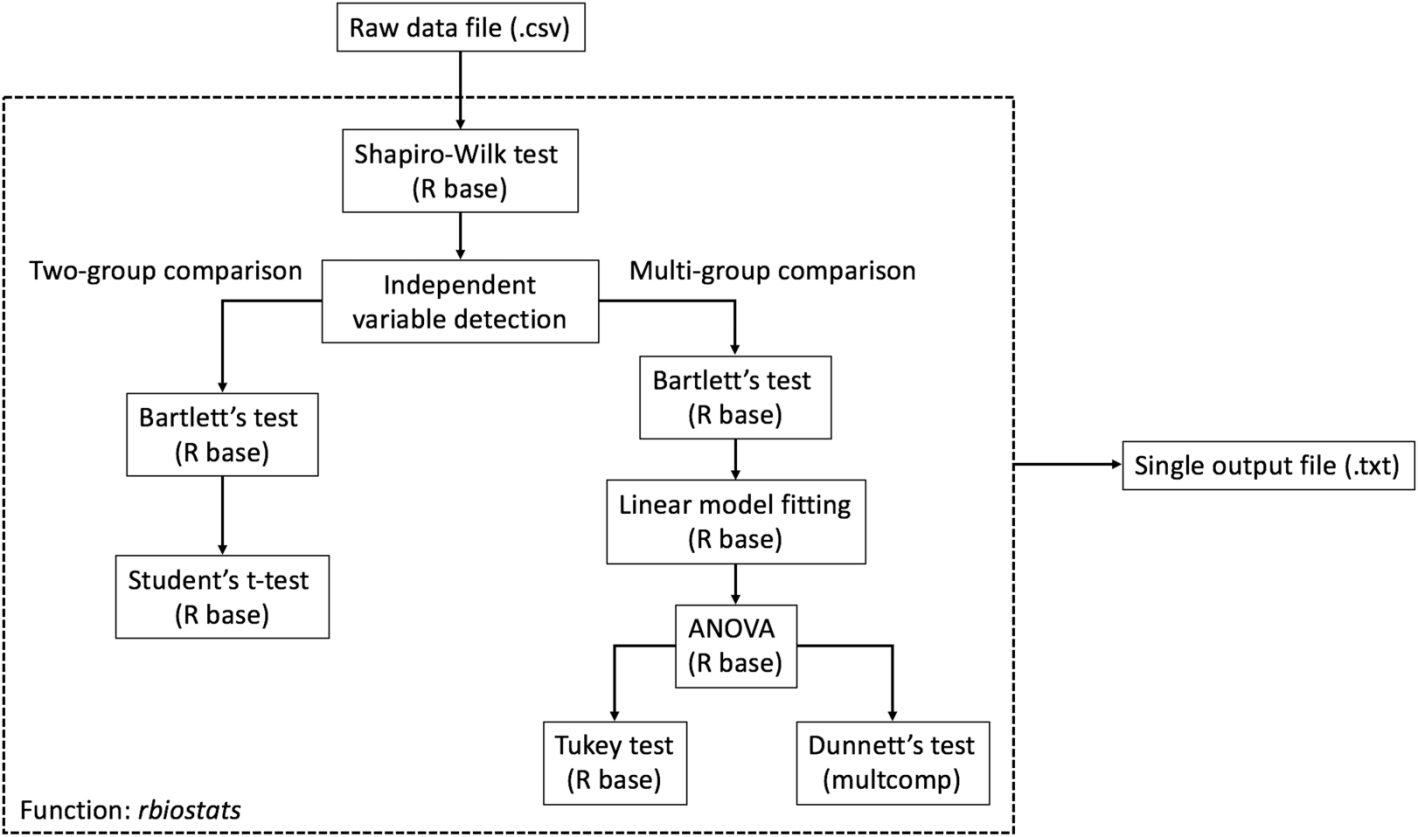
Workflow of the statistical analysis module. The core function rbiostats features Shapiro-Wilk normality test and Bartlett’s equal variance test as default analyses. Student’s t-test and ANOVA (with Tukey or Dunnett’s test) are included in the module.

The workflow of rbiostats function can be viewed in Fig. 1. It is worth noting that, for the same intended statistical test, the function is able to accept data files with more than one dependent variables and conduct the tests for each individual variable at the same time. Such configuration enables the capacity of processing grouped data sets according to certain criteria, e.g., grouping the data for genes from the same pathway. Upon designating the data file, rbiostats first conducts Shapiro-Wilk test to evaluate if the data for the replicates within the same independent variable follow a normal distribution. Then the Bartlett’s test of variance homogeneity is carried out. Upon both the normality and equal variance tests being performed, rbiostats function proceeds with the user-set statistical test of interest. The results for all the tests are written into a single results file (.txt) per input dataset.

We have also added independent variable detection and statistical test verification functionalities to the stats module. Specially, rbiostats function detects the total number of unique independent variables (without replicates, e.g., number of experimental groups) and verifies if the statistical test of choice is valid. For example, an error message and a suggestion of the correct tests will be written into the results file if Student’s t-test is set for a multi-group comparison where ANOVA with a post-hoc test is appropriate.

### Histogram module

**Figure 2:**
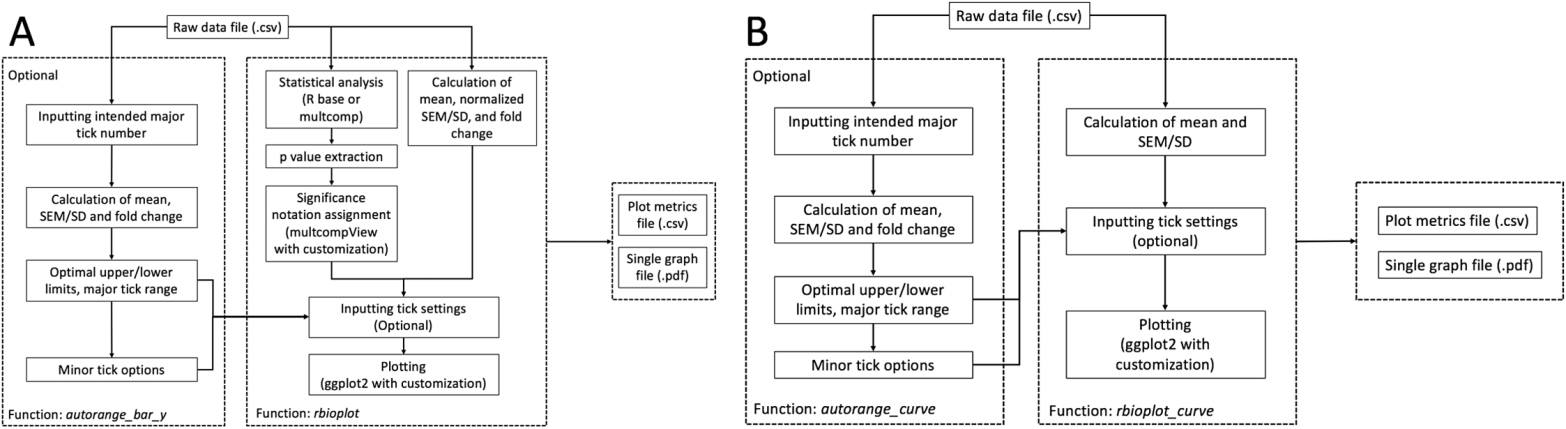
Workflow of the data visualization and axis range finding modules. (A) histogram module, and (B) joint-point curve module.

Histograms are commonly used to visualize and compare data in molecular biology and biochemistry lab techniques. Similar to the stats module, the histogram module is capable of processing and plotting grouped bar graphs in a single figure per data file. The popular R package ggplot2 serves as a foundation for the current module. Furthermore, we have incorporated extensive modifications and new functionalities into ggplot2 and the associated syntax to realize high data interpretability and, if needed, user customizability. The module is able to detect the number of dependent variables and adjust the aesthetic of the plot accordingly, with dependent variable(s) as the discrete value(s) for the x-axis. As a result, the core function rbioplot provides fully automated data manipulation, significance notation assigning based on the integrated statistical analysis, as well as interfaces for user-set graph title and axis labels. Figure 2A shows the workflow of the function. The module outputs a high quality image file (.pdf format, for size control) and a plot metrics file (.csv) as a reference.

For each dependent variable, the histogram module plots relative changes of the mean of the replicates comparing to the standard (e.g., the control group), with error bars representing either standard error of the mean (SEM) or standard deviation (SD) normalized against the mean of the standard (e.g., control group). Therefore, for each dependent variable, the data manipulation step involves calculation of the two aforementioned metrics, as well as fold changes relative to the standard. SEM is calculated using the equation:

$$SEM = {{standard\;deviation\;} \over {\sqrt n }}$$

where *n* is the number of replicates.

The automatic significance notation functionality is achieved by extracting p values from integrated statistical analysis. In general, fold change with a p < 0.05 is considered statistically significant, upon which a label appropriate to the statistical test of choice is assigned to the corresponding bar in the histogram. Specifically, the asterisk is used for both Student’s t-test and ANOVA with Dunnett’s test; whereas a lower case Roman alphabet is used for ANOVA with Tukey’s test. For Tukey’s test, the function multcompLetters from multcompView package is used to assign different letters to the group exhibiting statistically significant difference (Graves et al., 2015). The multcompLetter function automatically determines the letters based on the p values for each comparison alphabetically to the standard and independent variables based on the order of their appearance, i.e. the letter “a” is always given to the first variable in the Tukey results report. However, Tukey’s test in R base outputs results in a format optimized for easy interpretation. For example, “stress–control” will be written to the results file when comparing the variable “stress” to “control” in Tukey’s test. In such case, multcompLetter gives the letter “a” to “stress,” “b” to “control,” if the p value of the comparison is less than 0.05. Such behavior is problematic when plotting since the order of the letters is reversed in the histogram, for the layout of the bars representing the standard and independent variables usually follows the same order presented in the input data file, instead of the Tukey results report. To solve the issue, we have written a function revsort that reverses the display order of the standard and independent variables in a two-group comparison from Tukey’s test prior to executing multcompLetter, e.g., “stress–control” becomes “control–stress” in the Tukey results report after applying revsort function. Accordingly, the combination of revsort and multcompLetter has enabled rbioplot to automatically assign significance notations on the histogram for the Tukey’s test in the ideal order. Additionally, similar to rbiostats, independent variable detection and statistical test detection are also featured in the stats section of the histogram module.

Manipulation of right and left y-axis and minor tick functionalities are added to further improve the readability of the histogram. Given that currently ggplot2 only offers left-side y-axis, we incorporated custom-designed script to add the y-axis to the right-side of the graph. Specifically, selected functions from packages grid (part of R base) and gtable were used to copy and invert the mark position of the default left side y-axis to the right side (R Core Team, 2016; Wickham, 2016). Furthermore, since on-axis minor tick is unavailable in ggplot2 package, we have developed the following script as a workaround: Ticks with only blank (invisible) labels are considered as minor ticks and added in between the major ticks (i.e., ticks with visible labels). In the context of ggplot2 syntax, the y-axis labels are set based on the results of the script. Moreover, the tick range (interval of y-axis breaks) is calculated using following equation:

$$tick\;range = {{major\;tick\;range} \over {number\;of\;minor\;ticks-1}}$$

Given that the histogram module plots fold change of the mean, the default major tick range and number of minor ticks are set at 0.5 and 4. It is also worth noting that the upper limit of the y-axis is automatically set at maximum mean + SEM/SD, with ample space left for the inclusion of the significance notations. Furthermore, all the metrics for y-axis are fully user customizable if needed (see *axis range finding module* section). The package also includes a heatmap module with which users are able to produce simple heatmaps. The core function rbioplot_heatmap operates similarly as the histogram module and takes the exact same input file layout, with most of the arguments carried over from the core function rbioplot of the histogram module.

### Joint-point curve module

Joint-point curve is commonly seen when visualizing data with dependent variables representing a continuous relation, e.g., concentration series of an enzyme inhibitor. We have developed a joint-point plotting module for such purpose. Built upon the framework established in the histogram module, the core function of the current module rbioplot_curve takes the input file in the same format as other functions in the package. Instead of fold change, the function plots the mean with SEM/SD as the error bar (when needed) for each independent variable against the continuous dependent variables (i.e., x-axis values). Thus, SEM/SD values are not normalized based on the mean of the standard (e.g., control). Unlike the histogram, statistical analysis is not featured in the current module as significance notation is not required for the current purpose. The workflow of the function is similar to rbioplot (Fig. 2B), but without the fold change calculation, SEM/SD normalization or significance notation steps.

The rbioplot_curve function features default settings for major tick range and minor tick number for both x- and y-axes, with the most carried over from the histogram module. Specifically, major tick range and number of minor ticks for x-axis are set at 0.5 and 0 (no minor ticks), respectively; whereas the two metrics for y-axis are the same as the default settings for the histogram module. However, due to lack of data normalization, it is recommended to custom-set the values using the autorange_curve function from the axis range finding module as a guideline (see *axis range finding module* section). Similar to the histogram module, an image file (.pdf) and a plot metrics file (.csv) are generated by the joint-point curve function. The module is also capable of evaluating if the input data of interest requires manipulation (i.e., calculating mean and SEM/SD) and/or error bar for plotting. Specifically constructed for visualising standard and one or multiple independent variables, it is worth noting that only multi-curve plotting (i.e., more than one variable) is supported for the joint-point curve module in the current version (version 0.2.5).

### Axis range finding module

Functions autorange_bar_y and autorange_curve are developed for user-customizable axis range and tick setting functionalities for histogram and joint-point modules, respectively. Both functions share the same range finding algorithm. A general workflow for the range finding module is presented in Fig. 2A: Based on the user-set intended major tick number (excluding the first tick), the function processes the input data and generates the optimal major tick range with estimated lower and upper limit of the axis, as well as all the available minor tick number options.

Regarding data processing, both functions feature the same process from their counterparts in the plotting modules, i.e., autorange_bar_y calculates the fold change of the mean and normalized SEM/SD, whereas autorange_curve only focuses on mean and raw SEM/SD (when needed). The optimal major tick range of an axis is calculated using the equations below:

Let

$$raw\;major\;tick\;range = {{max-min} \over {intended\;major\;tick\;number}}$$

And

$$i = ceiling\left[ {lo{g_{10}}\left({raw\;major\;tick\;range} \right)} \right].$$

Thus

$$optimal\;major\;tick\;range = ceiling\left[ {{{raw\;major\;tick\;range/{{10}^i}} \over {0.05}}} \right] \times 0.05\; \times {10^i}$$

Estimated upper limit of the axis is calculated using the equation:

$$upper\;limit = optimal\;major\;tick\;range \times ceiling\left({{{max} \over {optimal\;major\;tick\;range}}} \right)$$

And the estimated lower limit of the axis is calculated through the following equation, if not using the default setting (zero):

$$lower\;limit = optimal\;major\;tick\;range \times floor\;\left({{{min} \over {optimal\;major\;tick\;range}}} \right)$$

For all the equations in the current section, *max* is the raw maximum value (fold change + normalized SEM/SD, or mean + SEM/SD); and *min* is the raw minimum value (the default is zero; otherwise, fold change—normalized SEM/SD, or mean—SEM/SD). It is worth noting that both floor and ceiling calculations follow the rounding rules specific to R. With the specific properties of raw data, the resulted upper and lower limits from the axis ranging finding module are only a close estimation for reaching the intended major tick number and may present slight discrepancies. As such, user can either use the range finding results directly for plotting, or utilize them as a guideline for fine-tuning when applying custom tick range settings in the plotting modules.

Furthermore, based on the rounded major tick range obtained above, we have developed an algorithm to automatically calculate all the minor tick options. In general, the following equation is used:

$$minor\;tick\;options = {{rounded\;major\;tick\;range} \over {divsors\;of\;rounded\;major\;tick\;range}}-1$$

As exhibited in the function above, the key step is to obtain all the divisors of the calculated major tick range. While it is quite straightforward to automatically determine all the divisors for an integer in R, doing so for decimal numbers requires a more complex process. The basic principle is to scale the decimal number up by a power of 10 to convert it to an integer, calculate the divisors, and then scale the results back using the same scaling factor. We have written an intermediate function all_dvsr to achieve such goal. Furthermore, a second scaling factor is included in the function to cope with the scenario where the maximum number of the potential minor ticks is less than four. For example, even if an integer, the major tick range “1” is applied with a scaling factor of 10 when calculating the divisor, so that decimal divisors are calculated for it, e.g., 0.1, 0.2, 0.5, etc. The divisor finding functionality is integrated in the autorange_bar_y and autorange_curve functions. Thus, no additional user input is required to acquire minor tick options. Accordingly, with the input data file and user-defined major tick numbers, both axis range finding functions output the estimated upper and lower limits, major tick range, and all minor tick options for the axis (or axes) of interest, which can be further used in their respective data visualization modules.

Given the unique characteristics of histogram and joint-point plots, specific adjustments are also made to the two functions in the current module. For example, autorange_bar_y only focuses on y-axis since range finding is only applicable to the y-axis for histogram; whereas autorange_curve operates on either or both axes as joint-point curve contain continuous values for the axes.

### Availability and installation

RBioplot is distributed under GPL-3 license through GitHub (https://github.com/jzhangc/git_R_STATS_KBS.git).The R package devtools (Wickham & Chang, 2016) is used to automatically install the package with all dependencies. After installing devtools package, run the following command devtools::install_github ("jzhangc/git_R_STATS_KBS/package/rbioplot") to install the package. An instruction, demo datasets and sample output files are available at http://kenstoreylab.com/?page_id=2448.

## Results and Discussion

For all case studies featured in this section, a working directory was designated using the R base function setwd. The input files were stored to the working directory prior to the analysis. The directory also served as the destination for all the output files generated by RBioplot.

### Case study 1: proteomic expression analysis (Western blotting)

We use part of the data from a study by Zhang & Storey (2012) to demonstrate the usage of the package for western blotting data analysis and visualization. In the study, western blotting was used to investigate the protein levels of cell cycle regulators in the liver tissue of wood frog (*Rana sylvatica*) under multiple stress conditions. Here we use RBioplot package to generate Fig. 6 from the original study.

The raw data were obtained as enhanced chemiluminescence (ECL) signals and Coomassie blue stained band intensities from the standardization protein bands. The dataset contains the protein level of several key cyclins under dehydration—rehydration cycle in the liver of the frogs. As such, the experimental conditions are considered as independent variables, while the individual cyclin proteins are the dependent variables. To prepare the input data file, we take the ratios of ECL and Coomassie intensities for all four cyclins tested for all animal groups, and then group them into a single data matrix (.csv). Table 1 shows the layout of the input data file. The first column is dedicated to the animal groups (independent variables), and the ratio values for the cyclins are presented starting from the second column. As shown in the same table, the input file contains the raw ratio values for all biological replicates. With the uneven number of biological replicates (*n* = 3–4) due to removal of outliers, the current dataset also demonstrates Rbioplot’s capability of automatically processing data files with missing values without additional data manipulation steps required from the user.

**Table 1:** Input file layout (.csv). (A) Data layout for the histogram module (case study 1). The first column is used for independent variables. Dependent variables start from the second column. The raw data points with biological replicates are presented. The current dataset also features missing values. (B) Data layout for the joint-point curve module (case study 2). A truncated version is shown due to the space limitation. The current dataset contains only one set of values for each animal group (i.e., no multiple replicates). The dependent variables (fraction number) show a continuous relation.

(A)				
Groups	Cyclin A	Cyclin B1	Cyclin D1	Cyclin E
Control	1	1	1	1
Control	1.19604093	0.709922771	0.829852824	1.121621622
Control		0.701239343	1.043122829	1.198614911
Control	1.090447809	0.713018827	1.086762242	
40% Dehydrated	0.653315462	0.243093923	0.159298618	
40% Dehydrated	0.92430544	0.615747952	0.169557985	0.611308901
40% Dehydrated	0.805826152	0.253410348	0.315173675	0.676607642
40% Dehydrated		0.510693792	0.313817882	0.665111318
Rehydrated	1.103239032	0.819114471	0.566761364	0.674198593
Rehydrated			0.687641907	0.757135246
Rehydrated	1.272757148	0.874822064	0.769900287	
Rehydrated	1.248098927	0.840052737	0.64319508	0.776602925

(B)				
Groups	5	6	7	8
Control	−0.031439236	−0.010101098	−0.005330851	0.113880783
40% Dehydration	0.016252368	−0.008646114	−0.008334101	−0.006418122

**Figure 3:**
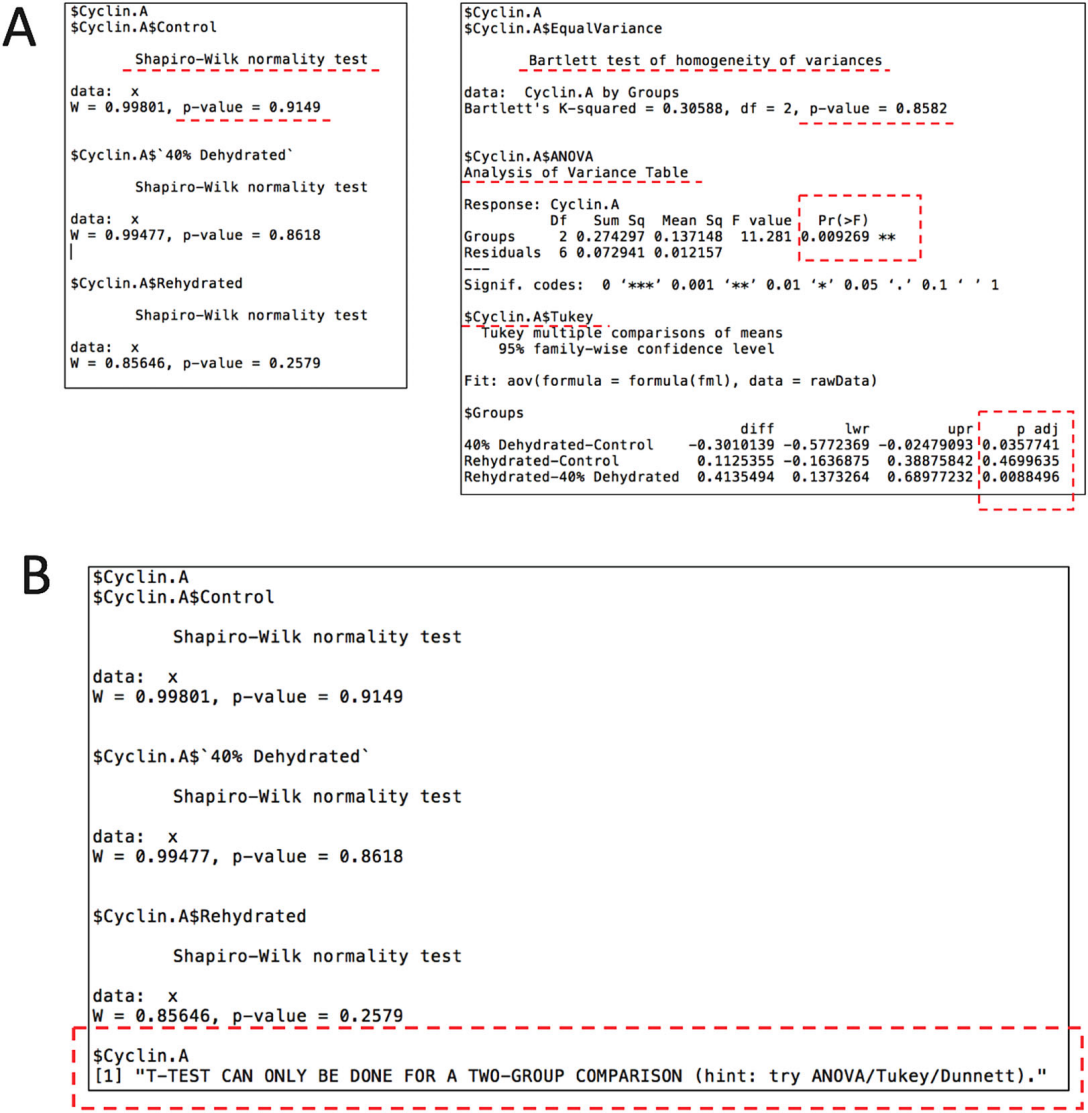
Example of the output file (.txt) by the stats module showing the results for cyclin A from case study 1 with the key sections highlighted. (A) The current file contains Shapiro-Wilk normality test, Bartlett’s equal variance test, ANOVA, and Tukey post-hoc test. (B) An example of the error message when setting Student’s t-test for a multi-group comparison.

Given that the current dataset contains a standard and two independent variables, an ANOVA with post-hoc test is appropriate. According to the original study, here we also use the Tukey post-hoc test to assess the changes of cyclins between all animal groups. To conduct statistical analysis, we use the following command: rbiostats("casestudy1.csv", Tp = "Tukey"). An output file (.txt) is then generated containing the all the stats results, i.e., Shapiro-Wilk normality test, Bartlett’s equal variance test, ANOVA, and Tukey’s test. Figure 3 shows the cyclin A portion of the report as an example. As seen in Fig. 3A, p values for both Shaprio-Wilk and Barlett’s tests are greater than 0.05, indicating normal distribution and equal variance, thereby suggesting the results of the upcoming ANOVA with Tukey’s test are valid. Figure 3B shows the error message when Student’s t-test is chosen for a multi-group comparison.

**Figure 4:**
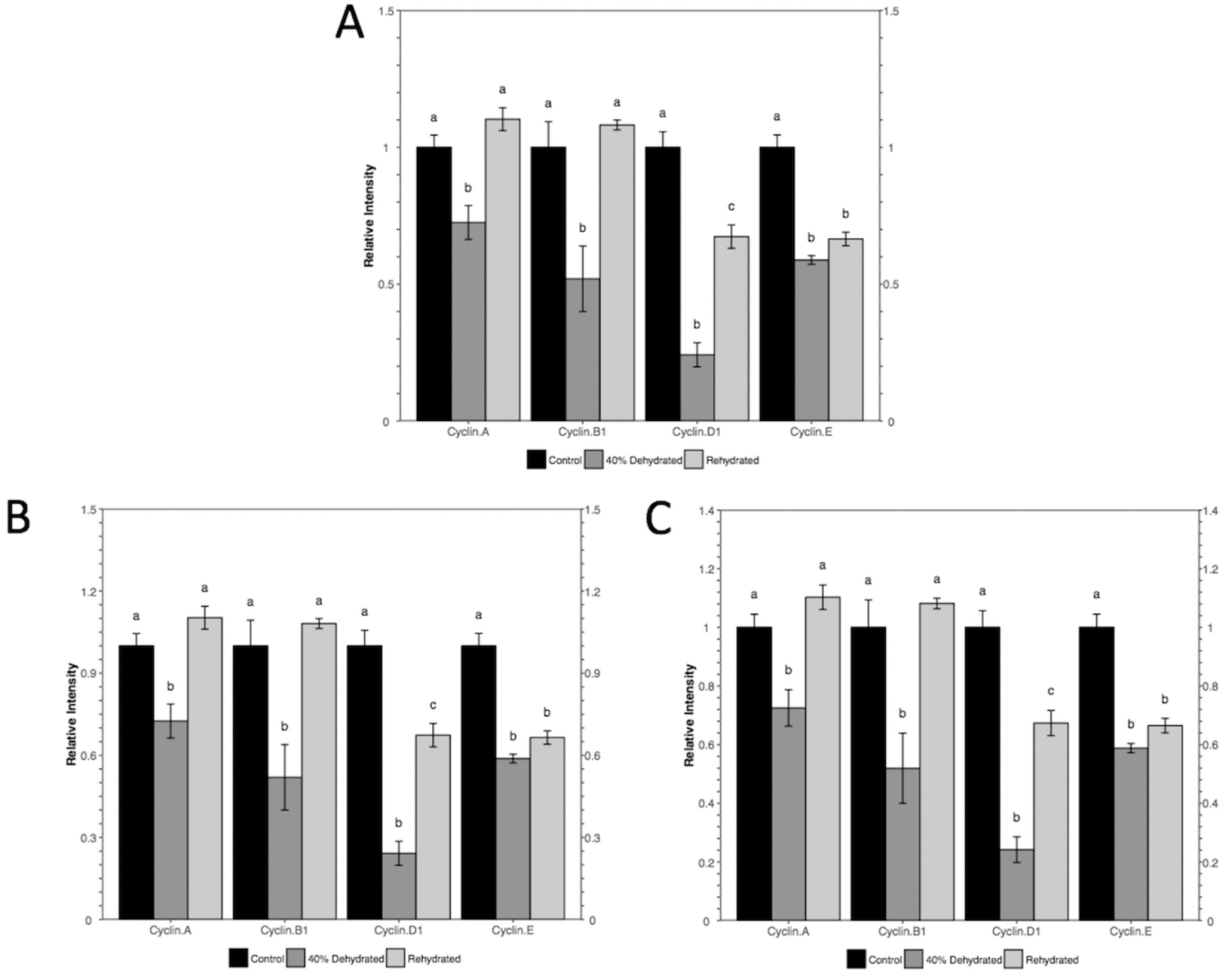
Histograms plotted using the histogram module (case study 1). The figure shows both (A) default and (B and C) custom tick settings, as well as automatic significance notation appropriate for Tukey post-hoc test (i.e., lower case Roman letters).

To visualize the data in the same fashion as the original study, the histogram module of the package is used with the following command: rbioplot("casestudy1.csv", Tp = "Tukey", yLabel = "Relative Intensity"). As shown in Fig. 4A, the resulting figure is consistent with the original study, only with different tick settings and more informative significance notations. Since the significance notation is automatically handled according to the imbedded stats function in the histogram module, we are able to achieve both accuracy of the label assignment and precise positioning on the graph. In addition to the image file, the function also generates a plot metrics file for reference (Table 2).

**Table 2:** Plot metrics for histogram. The file (.csv) is automatically generated upon running the plotting modules. For case study 1, the file includes experimental groups (Condition), cyclin targets (variable), normalized mean value using control as 1 (fold change) (NrmMean), normalized SEM (NrmSEM), and significance notations for ANOVA with Tukey’s test (Lbl).

Id	Condition	variable	NrmMean	variableSEM	NrmSEM	variableLbl	Lbl
1	Control	Cyclin.A	1	Cyclin.ASEM	0.044782409	Cyclin.ALbl	a
2	40% Dehydrated	Cyclin.A	0.725225991	Cyclin.ASEM	0.062004168	Cyclin.ALbl	b
3	Rehydrated	Cyclin.A	1.102725551	Cyclin.ASEM	0.041801495	Cyclin.ALbl	a
4	Control	Cyclin.B1	1	Cyclin.B1SEM	0.09349969	Cyclin.B1Lbl	a
5	40% Dehydrated	Cyclin.B1	0.51947888	Cyclin.B1SEM	0.119637014	Cyclin.B1Lbl	b
6	Rehydrated	Cyclin.B1	1.081452203	Cyclin.B1SEM	0.018013365	Cyclin.B1Lbl	a
7	Control	Cyclin.D1	1	Cyclin.D1SEM	0.056794487	Cyclin.D1Lbl	a
8	40% Dehydrated	Cyclin.D1	0.241896859	Cyclin.D1SEM	0.043813224	Cyclin.D1Lbl	b
9	Rehydrated	Cyclin.D1	0.673655355	Cyclin.D1SEM	0.042886507	Cyclin.D1Lbl	c
10	Control	Cyclin.E	1	Cyclin.ESEM	0.045240584	Cyclin.ELbl	a
11	40% Dehydrated	Cyclin.E	0.588219496	Cyclin.ESEM	0.015748309	Cyclin.ELbl	b
12	Rehydrated	Cyclin.E	0.664993817	Cyclin.ESEM	0.024568233	Cyclin.ELbl	b

While the default y-axis tick layout is adequate, custom-setting can also be achieved with or without the help of the axis range finding module. As an example, we set the intended major tick number at five. With the command autorange_bar_y("casestudy1.csv", nMajorTicks = 5), the estimated tick settings for reaching five major ticks are generated. With the default lower limit (zero), range finding module shows that the upper limit and major tick range of y-axis are 1.5 and 0.3, respectively. The results also suggest that eight minor tick options (i.e., 0, 1, 2, 4, 5, 9, 14, and 29) are supported by the current dataset with the custom tick settings. With these results, we can plot the data using the command: rbioplot("casestudy1.csv", Tp = "Tukey", yLabel = "Relative Intensity", y_custom_tick_range = TRUE, y_upper_limit = 2.8, y_major_tick_range = 0.4, y_n_minor_ticks = 5) (Fig. 4B). Furthermore, custom tick settings can be configured directly. For example, we can use the command rbioplot("casestudy1.csv", Tp = "Tukey", yLabel = "Relative Intensity", y_custom_tick_range = TRUE, y_lower_limit = 0, y_upper_limit = 1.4, y_major_tick_range = 0.2, y_n_minor_ticks = 4) to match the tick setting exactly as the original study, as shown in Fig. 4C.

### Case study 2: elution profile

For demonstrating joint-point plot, we use part of the data from a study that assesses the role of hexokinase in the dehydration tolerant African clawed frogs (*Xenopus laevis*) (Childers & Storey, 2016). Specifically, the joint-point curve module of the package is used to plot the data from Fig. 1 of the original study.

**Figure 5:**
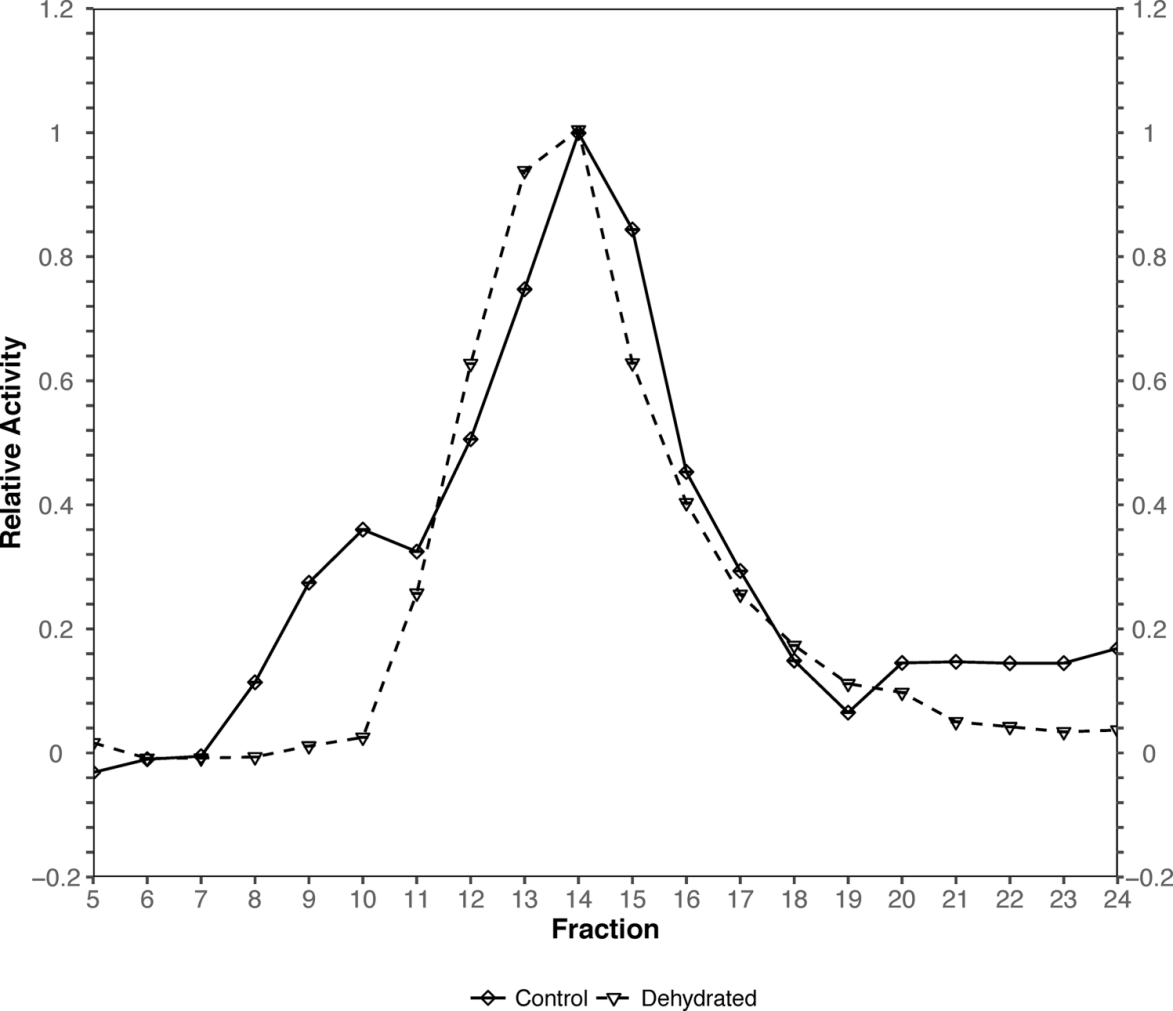
Joint-point curve plot (case study 2). The plot is produced by using the joint-pint curve module.

The original figure depicts the hexokinase elution profile in the skeletal muscle of both control and ∼40% dehydrated frogs using DEAE^+^ Sephadex column. The dataset contains hexokinase activity for 20 eluted fractions for both animal groups. We use the function rbioplot_curve to re-create the figure. Table 1 shows a truncated version of the input data file layout, with animal groups as independent variables and fractions as dependent variables. As shown in the table, the current data file only features a typical set of values for each animal group. In such case, joint-point curve module automatically detects and determines that the data can be plotted without error bar. By using the command rbioplot_curve("casestudy2.csv", x_custom_tick_range = TRUE, x_lower_limit = 1, x_upper_limit = 24, x_major_tick_range = 1, x_n_minor_ticks = 0, y_custom_tick_range = TRUE, y_lower_limit = −0.2, y_upper_limit = 1.2, y_major_tick_range = 0.2, y_n_minor_ticks = 4), the figure created from Rbioplot includes the same major tick range and lower/upper limits (0.2 and −0.2/1.2, respectively) of the original figure (Fig. 5). The resulted figure also includes both-side y-axis with minor ticks. Alternatively, axis range finding module can be used to further customize tick settings.

## Conclusion

The present work describes an R pipeline designed for comprehensive statistical analysis and data visualization. By dynamically integrating new functionalities and customizations to existing packages, RBioplot represents a fully automated and versatile data processing solution for molecular biology and biochemistry.

## Additional Information and Declarations

### Data Deposition

The following information was supplied regarding data availability: GitHub: https://github.com/jzhangc/git_R_STATS_KBS.git.
